# The Lattice Mismatch-Driven Photochemical Self-Assembly of Supported Heterostructures for Stable and Enhanced Electrocatalytic Carbon Dioxide Reduction Reaction

**DOI:** 10.3390/molecules29235560

**Published:** 2024-11-25

**Authors:** Yidan Liu, Xu Ren, Yali Ji, Ting Li, Rongrong Jia, Liyi Shi, Wenlong Zhou, Xiran Qiao, Lei Huang

**Affiliations:** 1 College of Textile Science and Engineering (International Institute of Silk), Zhejiang Sci-Tech University, Hangzhou 310018, China; liuyidan@zstu.edu.cn (Y.L.); 18868346307@163.com (X.R.); wzhou@zstu.edu.cn (W.Z.); 2 Key Laboratory of Functional Textile Material and Product, Ministry of Education, Xi’an Polytechnic University, Xi’an 710048, China; 3 Research Center of Nano Science and Technology, College of Sciences, Shanghai University, Shanghai 200444, China; r.jia@t.shu.edu.cn (R.J.); shiliyi@shu.edu.cn (L.S.); 4 Zhejiang Provincial Key Research Institute of Medical Materials and Tissue Engineering, Hangzhou 311121, China; 5 Laboratory of Advanced Materials, Department of Chemistry and Shanghai Key Laboratory of Molecular Catalysis and Innovative Materials, Fudan University, Shanghai 200438, China; 18113010001@fudan.edu; 6 Jiangxi Province Key Laboratory of Applied Optical Technology (2024SSY03051), School of Physical Science and Intelligent Education, Shangrao Normal University, Shangrao 334001, China; liting18@shu.edu.cn; 7 Department of Physics, Materials Genome Institute, Institute for Quantum Science and Technology, Shanghai University, Shanghai 200444, China; 8 School of Textile Science and Engineering, Xi’an Polytechnic University, Xi’an 710048, China

**Keywords:** photochemical self-assembly, lattice mismatch, heterostructure, electrocatalytic CO_2_ reduction reaction

## Abstract

Metallic heterostructural nanocrystals (HNCs) hold immense potential in electrocatalytic carbon dioxide reduction reaction (CO_2_RR) owing to their abundant active sites and high intrinsic activity. However, a significant challenge still remains in achieving controlled nucleation and growth sites for HNCs on supports and comprehending the influence of the structure–activity relationship on electrocatalytic CO_2_RR performance. This work presents a photochemical self-assembly technique without the necessity for reducing agents or facet-specific capping agents. By controlling lattice mismatch and manipulating transfer paths of photo-generated carriers, we can precisely direct the growth sites and nucleation of nanocrystals, enabling the self-assembly of supported core–shell and Janus nanostructures. Compared to Pd(T)@Au core–shell HNCs with the same loading, Pd cube–Au Janus HNCs exhibit significantly enhanced selectivity and stability toward carbon monoxide (CO) production in CO_2_RR at less negative potentials. The Pd cube–Au Janus HNC electrocatalyst achieved a Faradaic efficiency (FE) of 92.6 ± 3.5% for CO electroreduction, accompanied by a current density of 72.3 mA·cm^−2^ at −0.58 V. This work provides an effective strategy for designing advanced supported tandem electrocatalysts to boost the selectivity and durability test of CO_2_RR.

## 1. Introduction

Due to diverse active sites and efficient intrinsic activity, metallic heterostructural nanocrystals (HNCs) have shown excellent performance in catalysis and other fields, leading to their widespread applications [1,2,3]. In practical photocatalytic or electrocatalytic energy conversion applications, HNCs are typically integrated with supports to achieve the seamless integration of active components with electrodes and other devices [4,5,6]. Additionally, the confinement effect of the support effectively inhibits the migration and aggregation of HNCs, thereby maximizing the exposure of active sites [7,8]. This enhancement not only improves their electrochemical stability across a broad potential but also preserves the integrity of the crystal structure in various electrolytic environments [9,10]. Compared to the method of pre-synthesizing HNCs and subsequently loading them onto carbon black via physical adsorption [11], the in situ growth and assembly of HNCs on supports can significantly strengthen the interaction among the effective constituents and the support, thus greatly enhancing electron transfer or mass transport during catalysis and ultimately boosting the catalytic activity and stability [12,13,14]. However, the introduction of the support considerably complicates the nucleation and growth processes of the crystals [15]. Currently, significant challenges still remain in achieving the in situ growth and assembly of HNCs with specific morphologies, crystal phase structures, multicomponent compositions, and geometric distributions on the support [16]. Therefore, it is imperative to develop a research strategy for the scientific design and construction of HNCs on supports.

To achieve a green, efficient, low-carbon, environmentally friendly, and intelligent diversified development, it is crucial to provide material foundation and technological support for the energy technology revolution and clean resource utilization. At present, the wet chemistry synthesis method is among the most commonly efficient technologies in many well-known academic studies involving the macroscale preparation (supported) of HNCs [17]. Xia et al. controlled crystal growth by using reducing agents and facet-specific capping agents and realized the structural engineering regulation of crystals, thus improving their catalytic intrinsic activity and active site [18]. Xiong et al. achieved the small size and confinement regulation of crystals by using the hydrothermal method and crystal surface capping agents to composite crystals with nanosheets, which increased the density of active sites [19]. Zhang et al. investigated the phase engineering of nanomaterials (PENs), using the wet chemistry synthesis method and capping agents, based on epitaxial growth to achieve the phase engineering regulation of crystals with multi-chemical components, so as to improve the electrocatalytic performance [20]. However, these chemicals adhere to nanocrystals after preparation, thereby blocking the catalytic active sites. Removing residual reagents from the crystal surface will also lead to the formation of an oxide layer on the crystal surface and even damage the crystal structure, ultimately affecting the electrocatalytic activity [21]. Therefore, the developed research strategy needs to address this problem.

The photochemical deposition method is based on the photocatalytic mechanism and has advanced due to its green, efficient, and mild process, which has gained widespread favor. Its active ingredient has advantages such as strong controllability and great dispersion [22]. Electrocatalytic carbon dioxide reduction reaction (CO_2_RR) refers to an approach involving the conversion of CO_2_ into other high-value chemical substances through electrochemical reactions under the action of electrocatalysts [23]. Herein, we propose a photochemical self-assembly technique to effectively limit the influence of reducing agents, facet-specific capping agents, and other chemical agents, and we investigate the nucleation growth sites and different structures of bimetals on reduced graphene oxide (rGO) to enhance the impact of electrocatalytic CO_2_RR performance. By controlling lattice mismatch and manipulating transfer paths of photo-generated carriers, the nucleation growth sites and the reduction paths of HNCs can be precisely controlled to realize the self-assembly growth process of core–shell and Janus-structure HNCs on the support. Particularly, the obtained HNCs can be used directly without post-treatment and the addition of carbon black as a support. Compared to Pd(T)@Au core–shell HNCs with the same loading, Pd cube–Au Janus HNCs exhibited significantly enhanced selectivity and stability toward carbon monoxide (CO) production in CO_2_RR at less negative potentials. Pd cube–Au Janus HNCs exhibited better performance for the CO_2_-to-CO reduction, with an outstanding CO Faradaic efficiency (FE) of 92.6 ± 3.5% and current density of CO up to 72.3 mA·cm^−2^ at −0.58 V, as well as a total current density of 1.8 A·cm^−2^ at −1.78 V. These findings provide insights into the design and synthesis of supported heterostructure nanocrystals to enhance their activity in various electrocatalytic adhibitions.

## 2. Results and Discussion

### 2.1. Synthesis and Characterization of Supported Pd(T)@Au and Pd Cube–Au HNCs

The mechanism of photochemical deposition is as follows: When a nanometer semiconductor absorbs photons with higher energy than band gap energy under light irradiation, the electrons on its valence band (VB) are excited, inducing their transition to its conduction band (CB), thereby generating free-moving photo-generated electrons (*e*^−^) while leaving photo-generated holes (*h*^+^) on the valence band. Electrons transitioning to the conduction band migrate toward the bulk surface, thereby reducing the metal precursor and achieving the photochemical deposition of metal crystals [24]. The generalized equation of metal M photochemical deposition is expressed using Equation (1):

M^n+^(aq) + ne^−^ → M(s) (1)

**Figure 1:**
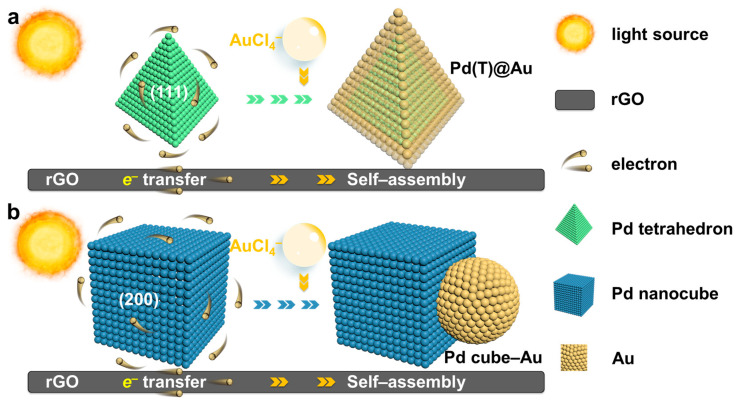
Schematic illustration of the photochemical self-assembly paths of (**a**) Pd(T)@Au and (**b**) Pd Cube–Au HNCs on rGO support.

The conceptual framework of the process involves the photochemical deposition of bimetallic HNCs (M_A_, M_B_) onto the support, encompassing macroscopic preparation and microscopic self-assembly processes, as illustrated in Figure S1 and Figure 1, respectively. Initially, nanosemiconductors, e.g., TiO_2_; conductive reservoirs, e.g., rGO; and sacrificial reagents, e.g., MeOH were uniformly mixed to form hybrid supports through van der Waals forces. Due to the fact that oxygen can participate in the reduction of metal precursors to form metal oxides, thereby affecting the formation and catalytic effect of HNCs, a saturated inert gas Ar is required throughout the photochemical self-assembly process to protect the self-assembly of HNCs. In the first step of the photochemical reaction, photo-generated carriers generated by the nanometer semiconductor removed the oxygen-containing functional groups from the conductive reservoir while regulating the defect engineering of the reservoir, thus improving the electron transport ability and mass transfer performance [25] and forming a composite support. In the second step, {111}-terminated Pd tetrahedron (Pd(T)) and Pd nanocube (Pd cube) with (200) crystal plane seeds as reference nanocrystals were initially reduced on rGO support through photodeposition based on our previous work [26,27]. Subsequently, we meticulously controlled the reduction paths to facilitate the deposition of Au onto the surfaces of supported Pd seeds and investigated the deposition sites of Au nanocrystals through lattice mismatch in the third step. Our findings indicate that within the same liquid-phase environment and metal content, as detected by inductively coupled plasma–optical emission spectrometer (ICP-OES), the binding modes of Au nanocrystals to Pd(T) and Pd cubes exhibited a stark contrast. The detailed preparation processes are provided in Materials and Methods of Supplementary Materials. A comparison of the morphologies and structures was performed using transmission electron microscopy (TEM), high-resolution transmission electron microscopy (HRTEM), and energy dispersive spectroscopy (EDS).

**Figure 2:**
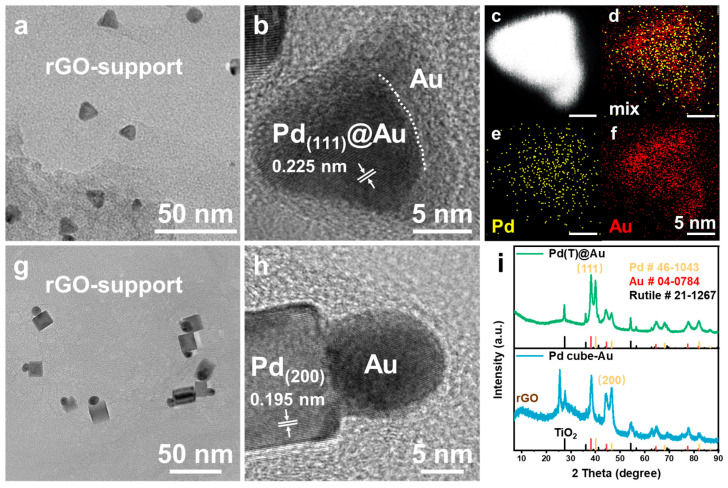
Characterization of Pd and Au in two photochemical deposition modes: (**a**) TEM, (**b**) HRTEM, and (**c**) HAADF-STEM images; (**d**) Pd, Au overlap; (**e**) Pd and (**f**) Au EDS elemental mapping images of supported Pd(T)@Au HNCs; (**g**) TEM and (**h**) HRTEM images of supported Pd Cube–Au HNCs; (**i**) XRD patterns of supported Pd(T)@Au and Pd Cube–Au HNCs.

As shown in Figure 2a–f, TEM and HRTEM results indicate that HNCs still exhibited tetrahedral morphology after Au deposition on the Pd(T) surface. EDS elemental mappings clarify that Au nanocrystals and Pd(T) formed a core–shell structure, denoted as Pd(T)@Au (the abbreviated names are listed in Table S1). Interestingly, Au nanocrystals were deposited only on one face of the Pd cube, as shown in Figure 2g,h, forming a Janus structure (Janus structure is a special structure composed of two or more components with different physical properties [28]), denoted as Pd Cube–Au. The X-ray diffraction (XRD) patterns indicate that the Au nanocrystals produced by photochemical deposition were mainly enclosed by {111} facets (Figure 2i). Based on the PDF cards for Palladium (syn, PDF#46-1043) (Figure S2) and Gold (syn, PDF#04-0784) obtained from XRD, the lattice constant of *d*_Pd(111)_ was 0.225 nm, while the lattice constant of *d*_Pd(200)_ was 0.195 nm, and that of *d*_Au(111)_ was 0.236 nm, which align with the (HR)TEM results presented in Figure S3. Utilizing Equation (2) for lattice mismatch calculations, it can be concluded that the lattice mismatch between Au deposited on {111}-terminated Pd(T) ranged from 4.7% to 4.9%, whereas the mismatch observed on Pd with respect to its (200) crystal planes reached as high as 17.4% to 21.0% (Table 1). Therefore, a small lattice mismatch is prone to in situ epitaxial growth and the formation of core–shell structures, while a larger lattice mismatch leads to the formation of Janus structures or even M_A_ support and M_B_ structures [29,30].

(2) $$Lattice\;mismatch\;\left(\%\right)=\frac{\left|d_{M_{A\left(hkl\right)}}-d_{M_{B\left(hkl\right)}}\right|}{d_{M_{A\left(hkl\right)}}}\times 100\%\;\sim \;\frac{\left|d_{M_{A\left(hkl\right)}}-d_{M_{A\left(hkl\right)}}\right|}{d_{M_{B\left(hkl\right)}}}\times 100\%$$

where $d_{M_{A\left(hkl\right)}}$ and $d_{M_{B\left(hkl\right)}}$ are the lattice spacing under (*h k l*), respectively.

**Figure 3:**
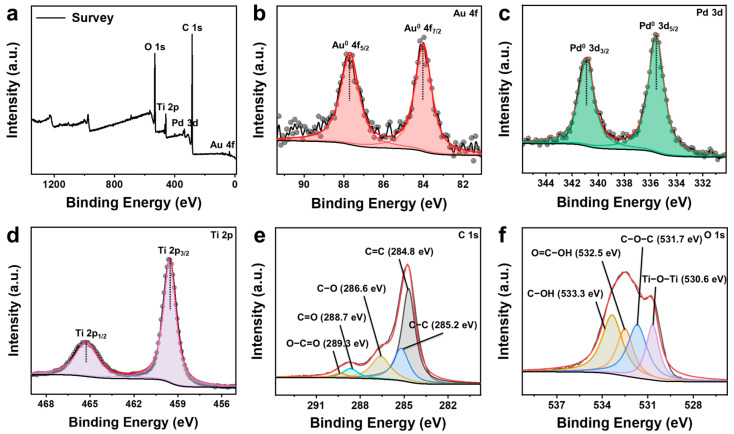
XPS spectra: (**a**) XPS survey; high-resolution (**b**) Au 4f, (**c**) Pd 3d, (**d**) Ti 2p, (**e**) C 1s, and (**f**) O 1s core-level XPS spectra of supported Pd(T)@Au and Pd Cube–Au.

As shown in Figure S4, there is no outline of the oxygen element, indicating the absence of metal oxide formation. Through X-ray photoelectron spectroscopy (XPS) testing, we found that the Pd(T)@Au and Pd Cube–Au valence states were consistent; therefore, we selected one of the composite elemental chemical states for analysis. As shown in the XPS survey spectra, characteristic peaks of Pd, Au, Ti, C, and O were observed in TiO_2_, rGO, Pd seeds, and Au nanocrystals (Figure 3a). From the high-resolution Pd 3*d* and Au 4*f* core-level XPS spectra, it can be observed that Pd and Au mainly exist in metallic states (Pd^0^ and Au^0^) as zero-valent states, as evidenced by the peaks of the Au element at 4*_f_*_5/2_ (87.7 eV) and 4*f*_7/2_ (84.0 eV) (Figure 3b) and typical peaks of 3*d*_3/2_ (340.8 eV) and 3*d*_5/2_ (335.6 eV) of the Pd element (Figure 3c), which could eliminate the difference in valence states and provide a stable foundation. The semiconductor TiO_2_ did not participate in the reaction and solely provided electrons; consequently, no alteration was observed in the 2*p*_1/2_ (465.2 eV) and 2*p*_3/2_ (459.5 eV) states of the Ti element (Figure 3d) [31]. The high-resolution deconvolution C 1*s* spectra for both samples reveal five distinct peaks, two at 284.8 and 285.2 eV assigned to the C=C and C−C bond of pure carbon, while the remaining peaks at 289.3, 288.7, and 286.6eV are attributed to O−C=O, C=O and C−O functionalities, respectively (Figure 3e). Furthermore, the high-resolution deconvolution O 1*s* spectra reveal four characteristic peaks as follows: Ti−O−Ti (530.6 eV), C−O−C (531.7 eV), O=C−OH (532.5 eV), and C−OH (533.3 eV), respectively (Figure 3f). The vibrational characteristics of carbon and oxygen primarily originate from rGO supports, whereas those of titanium and oxygen predominantly arise from TiO_2_.

### 2.2. Self-Assembly Mechanism of Photochemical Deposition

**Figure 4:**
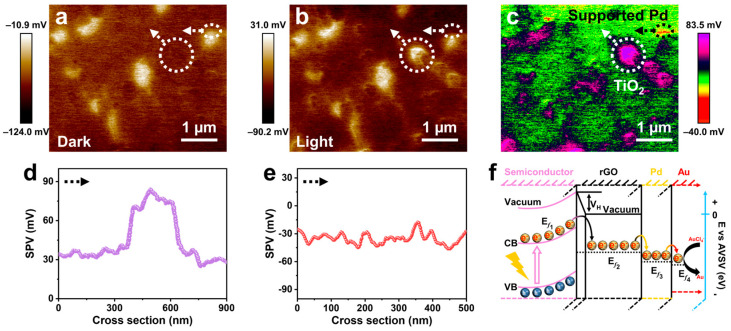
Distribution of photo-generated charge: surface potential images in the (**a**) dark state and (**b**) under the light; (**c**) SPV images of TiO_2_-rGO-supported Pd seeds; SPV signals of (**d**) TiO_2_ and (**e**) supported Pd measured along the arrow line marked in (**c**); (**f**) schematic diagram of the electron transfer mechanism.

To further understand the photochemical deposition process, we investigated the self-assembly mechanism of HNCs on supports. The reduction of metal precursors relies on photo-generated electrons, so it is crucial to clarify the dynamic distribution of photo-generated electrons. We conducted a surface photovoltage microscopy (SPVM) platform to visualize the nanoscale distribution of photo-generated charges, thereby gaining deeper insights into their migration paths [32]. The magnitude of the surface photovoltage (SPV) serves as an indicator for the spatial separation of photo-generated charges resulting from photoinduced excitation and transport behaviors [33]. The detailed amplitude modulation–kelvin probe force microscopy (AM-KPFM) measurements are outlined in Materials and Methods of Supplementary Materials. Under illumination, holes and electrons in the materials began to separate. By correlating atomic force microscopy (AFM) topography (Figure S5), we derived profiles across the TiO_2_-rGO-supported Pd seeds by subtracting the potential measurements taken under dark conditions (Figure 4a) from those obtained under 380 nm solar light irradiation (Figure 4b). The SPV image (Figure 4c) illustrates the spatial distribution of photon-generated carriers, with morphological features represented through distinct regions and color coding that indicates the magnitude of surface potential. Figure 4d,e quantitatively illustrate the SPV distributions for TiO_2_- and rGO-supported Pd seeds, respectively, derived from Figure 4c. The region supporting Pd, denoted by white circles, appears in red, signifying a negative SPV signal indicative of electron accumulation on Pd; conversely, the TiO_2_ region marked with white circles exhibits a purple hue, representing a positive SPV signal associated with hole aggregation under illumination excitation. From the perspective of potential gradients (Figure 4f), it can be inferred that photo-generated electrons from TiO_2_ may migrate to rGO-supported Pd, thereby facilitating a reduction reaction involving metal precursors [34].

The charge of each component can also affect the growth sites of crystals. Based on the principle of repulsion between identical charges and attraction between opposite charges [35], the pH environment of the solution can alter the assembly process of HNCs. At the end of step 2 of the photochemical reaction, as shown in Table S2, the initial solution containing supported Pd seeds had a pH value of approximately 5.0. At this point, Au nanocrystals were reduced to Pd seeds. When we adjusted the pH to 2.0 at this time, it was observed that the zeta potential of the solution became positive when the pH value dropped below 3.0. Due to the negative charge of the chloroauric acid ligand [AuCl_4_]^−^, it is easy to combine with the positive charge on the rGO support surface and reduce to rGO instead of Pd seeds through electrons, forming Au/Pd(T) and Au/Pd cube structures, respectively (Figure S6a,b). The change in zeta potential caused by pH value altered the nucleation and growth sites of crystals. As shown in Figure S6c,d, individual Au nanocrystals can form tetrahedrons, further indicating that the substrate repels the Au precursor to reduce to rGO and self-form spherical structures in the liquid phase. In summary, the transfer path of electrons, the regulation of zeta potential, and the lattice mismatch between crystals collectively contribute to the formation of the oriented self-assembly of HNCs.

### 2.3. Electrocatalytic CO_2_RR Performance

**Figure 5:**
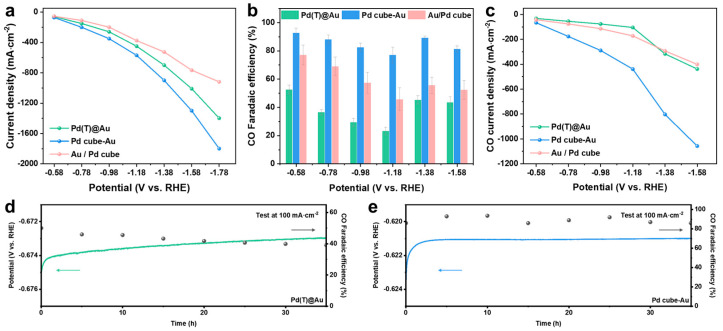
Electrocatalytic CO_2_RR performance: (**a**) total current densities; (**b**) CO FEs and (**c**) CO current densities (partial current density) on supported Pd(T)@Au, Pd Cube–Au, and Au/Pd cube HNCs at different fixed potentials (−0.58~−1.78 V vs. RHE); potential versus time of (**d**) Pd(T)@Au and (**e**) Pd Cube–Au HNCs at 100 mA·cm^−2^ constant current density and corresponding CO FEs.

The different self-assembly modes of Pd and Au have a significant impact on the electrocatalytic CO_2_RR performance [36]. We evaluated the electrocatalytic CO_2_RR performance and the corresponding linear sweep voltammetry (LSV) curves of the supported Pd(T)@Au core–shell structure, Pd Cube–Au Janus structure, and unstructured Au/Pd cube HNCs to further understand the catalytic activity, selectivity, and mechanism of electrochemical CO_2_RR in the flow cell. As shown in Figure 5a and Table S3, all samples exhibited super-high current density at different fixed potentials, particularly the Pd Cube–Au Janus structure, which had a total current density exceeding 1 A·cm^−2^ at −1.45 V (versus reversible hydrogen electrode, RHE), reaching industrial benchmarks [37]. Figure S7 shows that the main products of the Pd(T)@Au core–shell structure were CO and CH_4_, the maximum CO FE at −0.58 V was 52.6 ± 3.8%, and the maximum CH_4_ FE at −0.78 V was 36.4 ± 0.7%, accompanied by the conversion of some trace final products such as EtOH, acetate, HCOOH, C_2_H_4_, H_2_, etc. The CO final product selectivity of the Pd Cube–Au Janus structure was the highest compared with that of Pd(T)@Au and Au/Pd cube HNCs at various potentials, as shown in Table S4. Faradaic Efficiency (FE) refers to the ratio of the amount of electrocatalytic final products generated in actual electrochemical reactions to the theoretical products generated. It reflects the efficiency of the current used to generate chemical products in electrochemical reactions [38]. Moreover, the CO_2_RR conversion FEs of both the Pd Cube–Au Janus and Pd(T)@Au core–shell structures could reach 100 ± 3% at −0.58 V (RHE).

A normalization comparison of CO FE potential revealed that the FE of the Pd Cube–Au Janus structure could reach 92.6 ± 3.5% at −0.58 V (Figure 5b and Table S4). The calculation also shows that the CO current density of the Pd Cube–Au Janus structure was also the highest among the three at different potentials, which could reach 1056.9 mA·cm^−2^ at −1.58 V (Figure 5c and Table S5). The electrochemical stability of Pd(T)@Au core–shell HNCs and the Pd Cube–Au electrocatalyst was tested under 100 mA∙cm^−2^ constant current density over 35 h. Compared to Pd(T)@Au, Pd Cube–Au demonstrated a better steady voltage region and a higher FE for CO throughout the entire process, and there was no decreasing trend in the potential. The Faraday efficiency of CO still reached over 80% after 35 h (Figure 5d,e). This could be attributed to the higher electrocatalytic stability of (200)-facet-enclosed HNCs compared to (111)-facet-enclosed HNCs. Despite the same elemental mass ratio and series structures (both of Janus and core–shell structures), the change in the catalyst structure in tandem altered the key steps of CO_2_RR-to-CO, leading to changes in selectivity, as well as simultaneous catalytic activity and stability enhancement [39,40,41].

## 3. Conclusions

In summary, we investigated the regulation of the nucleation and growth sites of crystals by optimizing lattice mismatch and manipulating the transfer pathways of photo-generated carriers on supports through a photochemical self-assembly approach that is free from reducing agents and facet-specific capping agents. This method effectively governs the formation mechanisms of core–shell structures and Janus structures on these supports. Compared to Pd(T)@Au core–shell HNCs with equivalent loading, Pd Cube–Au Janus HNCs demonstrated significantly improved selectivity and stability for CO production in CO_2_RR at less negative potentials. The Pd Cube–Au Janus HNC electrocatalyst achieved a high FE of 92.6 ± 3.5% for CO electroreduction, accompanied by a current density of 72.3 mA·cm^−2^ at −0.58 V. All samples exhibited exceptionally high current densities, particularly the Pd Cube–Au Janus structure, whose total current density exceeded 1 A·cm^−2^ at −1.45 V, thereby reaching industrial benchmarks. This research provides an effective strategy for designing advanced supported tandem catalysts aimed at enhancing both selectivity and stability in electrocatalysis.

## Figures and Tables

**Table 1:** Lattice mismatches between Pd(T)/Pd cube and Au with enclosed {111} facets.

M_A_	M_B_	Lattice Mismatch (%)
Pd(T)_(111)_	Au_(111)_	4.7~4.9
Pd cube_(200)_	Au_(111)_	17.4~21.0

## Data Availability

Data are contained within the article and the Supplementary Materials.

## Supplementary Materials

The following are available online at https://www.mdpi.com/article/10.3390/molecules29235560/s1, Materials and Methods; Figure S1: Schematic illustration of the preparation process; Figure S2: XRD pattern of supported Pd(T) HNCs.; Figure S3: TEM and HRTEM of (a,b) supported Pd (T) and (c,d) Pd cube; Figure S4: EDS elemental mapping images of (a) C, (b) O, and (c) atomic ratio EDS spectra of Pd(T)@Au; Figure S5: AFM topography of supported Pd seeds; Figure S6: TEM images of supported (a) Au/Pd cube and (b) Au/Pd(T). (c) TEM and (d) HRTEM of supported Au; Figure S7: Average FEs of CO_2_RR products over (a) Pd(T)@Au and (b) Pd Cube–Au at different potentials; Table S1: Abbreviation names of the samples; Table S2: Zeta potentials of supported Pd seeds at different pH values in solution; Table S3. Total current densities on supported HNCs at different fixed potentials; Table S4. CO FEs on supported HNCs at different fixed potentials. Table S5. CO current densities (partial current density) on supported HNCs at different fixed potentials; Refs. [27,42,43] are cited in Supplementary Materials.
