# Supplementary material for: The Lattice Mismatch-Driven Photochemical Self-Assembly of Supported Heterostructures for Stable and Enhanced Electrocatalytic Carbon Dioxide Reduction Reaction
Source: Molecules. 2024 Nov 25;29(23):5560. doi: 10.3390/molecules29235560 (PMC11643941; doi:10.3390/molecules29235560)
Supplement: Supplementary file 1 [file molecules-29-05560-s001.zip › molecules-3315876-supplementary.pdf]

Supplementary Materials

Lattice mismatch-driven photochemical self-assembly of supported heterostructures for stable and enhanced electrocatalytic carbon dioxide reduction reaction

Yidan Liu [1], Xu Ren [1,4], Yali Ji [5], Ting Li [6], Rongrong Jia [3,7], Liyi Shi [3], Wenlong Zhou [1], Xiran Qiao [2,8,*] and Lei Huang [3,*]

1 College of Textile Science and Engineering (International Institute of Silk), Zhejiang Sci-Tech University, Hangzhou 310018, People's Republic of China

2 Key Laboratory of Functional Textile Material and Product, Ministry of Education, Xi'an Polytechnic University, Xi'an, People's Republic of China 710048

3 Research Center of Nano Science and Technology, College of Sciences, Shanghai University, Shanghai 200444, People's Republic of China

4 Zhejiang Provincial Key Research Institute of Medical Materials and Tissue Engineering, Hangzhou, 311121, People's Republic of China

5 Laboratory of Advanced Materials, Department of Chemistry and Shanghai Key Laboratory of Molecular Catalysis and Innovative Materials, Fudan University, Shanghai 200438, People's Republic of China

6 Jiangxi Province Key Laboratory of Applied Optical Technology (2024SSY03051), School of Physical Science and Intelligent Education, Shangrao Normal University, Shangrao 334001, People's Republic of China

7 Department of Physics, Materials Genome Institute, Institute for Quantum Science and Technology, Shanghai University, Shanghai 200444, People's Republic of China

8 School of Textile Science and Engineering, Xi'an Polytechnic University, Xi'an, People's Republic of China 710048

* Correspondence: qiaoxiran@xpu.edu.cn (X.Q.); leihuang@shu.edu.cn (L.H.)

Table of Content

Supplementary Materials 1
Table of Content 2
Materials and Methods 3
Supporting Figure S 6

Figure S1. Schematic illustration of the preparation process. 6

Figure S2. XRD pattern of supported Pd(T) HNCs. 7

Figure S3. TEM and HRTEM of (a,b) supported Pd(T) and (c,d) Pd cube. 8

Figure S4. EDS elemental mapping images of (a) C, (b) O, and (c) atomic ratio EDS spectra of Pd(T)@Au. 9

Figure S5. AFM topography of supported Pd seeds. 10

Figure S6. TEM images of supported (a) Au / Pd cube and (b) Au / Pd(T). (c) TEM and (d) HRTEM of supported Au. 11

Figure S7. Average FEs of CO_2RR products over (a) Pd(T)@Au and (b) Pd cube-Au at different potentials. 12

Supporting Tables 13

Table S1. Abbreviation names of the samples. 13

Table S2. Zeta potentials of supported Pd seeds at different pH values in solution. 14

Table S3. Total current densities on supported HNCs at different fixed potentials. 15

Table S4. CO FEs on supported HNCs at different fixed potentials. 16

Table S5. CO current densities (Partial current density) on supported HNCs at different fixed potentials. 17

References 18

Materials and Methods

Materials

All chemicals were used as received without further purification. Tetraamminepalladium(II) dichloride ($Pd(NH_3)_4Cl_2 \cdot H_2O$, ≥ 99%) was purchased from Aladdin. Titanium (IV) oxide (TiO_2) (Rutile, < 100 nm) and Nafion solution (5.0%) were purchased from Sigma-Aldrich. Hydrogen tetrachloroaurate tetrahydrate ($HAuCl_4 \cdot 4H_2O$, ≥ 99.9%), absolute methanol (MeOH), phosphoric acid (H_3PO_4, 85%), concentrated sulfuric acid (H_2SO_4, guaranteed reagent, 98%), hydrogen peroxide (H_2O_2, 30%), hydrochloric acid (HCl, 35~37%), cetyltrimethylammonium bromide (CTAB) (⩾99%, Acros) and other chemical reagents were purchased from Sinopharm Chemical Reagent Company. High purity flake graphite (XF010-1) was purchased from XFNANO. The ultrapure (UP) water (ρ＞18.25 MΩ·cm) was used in all experiments.

Preparation of graphene oxide (GO) solution

A mixture of H_2SO_4 and H_3PO_4 with a volume ratio of 120:13.33 mL was added to a three-necked flask containing 1.0 g graphite flakes in an ice bath and then followed by the slow addition of 6.0 g $KMnO_4$. The temperature of bath was subsequently raised to 50 °C and continuously stirred for 12 h. Following this, added a solution consisting of 150.0 mL ultrapure (UP) water and 1.5 mL H_2O_2 (30%), the resulting mixture was washed with 5%vol HCl solution and then subjected to centrifugation at 12000 rpm for 15 min, removed the supernatant after centrifugation, added UP water to wash and centrifuge at 12000 rpm for 15 min again and repeated three times. Finally, the residual solid was vacuum-dried at 60 ℃ and then dispersed in UP water to obtain GO solution [S1].

Synthesis of TiO_2-GO-supported Pd(T)@Au

Initially, 1 mg of TiO_2 was subjected to ultrasonic treatment in 250 mL of ultrapure water (UP) water for a duration of 10 minutes. Subsequently, 1.88 mL GO solution (8 mg/mL) and 10 mL absolute methanol were added to form TiO_2-GO. The resulting mixture was continuously purged with Ar and stirred throughout the entire preparation process in the photoreactor and then illuminated by a mercury lamp at (130 V*4 A) W (130 mW/cm^2) for 15 min, resulting in the formation of TiO_2-rGO (1) support. After turning off the lamp, 315.0 μL $Pd(NH_3)_4Cl_2 \cdot H_2O$ (10 mg/mL) (1.25 mg Pd element, equivalent to 7.0 wt% Pd loading) was added and followed by irradiation from a mercury lamp at (130 V*5 A) W (210 mW/cm^2) for 30 min, supported Pd tetrahedron (Pd(T)) seeds were formed on rGO (TiO_2-GO-supported Pd). On the basis of synthesis of TiO_2-rGO-supported Pd(T) seeds, turned off the lamp after the irradiation, 112.6 μL $HAuCl_4 \cdot 4H_2O$ (100 mg/mL) (0.54 mg Au element, equivalent to 3.0 wt% Au loading) was added, and then the solution was further illuminated by a mercury lamp at (130 V*5 A) W (210 mW/cm^2) for 30 min, and followed by a mercury lamp at (130 V*8 A) W (360 mW/cm^2) for 90 min. The black solid of Pd(T)@Au HNCs was collected through a filtration and drying process.

Synthesis of GO-supported Pd cube-Au

Initially, 1 mg of TiO_2 and 1.88 mL GO solution (8 mg/mL) were added to 250 mL of UP water dispersed uniformly by ultrasound. The resulting mixture was continuously purged with Ar and stirred throughout the entire preparation process in the photoreactor and then illuminated by a mercury lamp at (130 V*4 A) W (130 mW/cm^2) for 15 min, resulting in the formation of rGO (1) support. After turning off the lamp, 315.0 μL $Pd(NH_3)_4Cl_2 \cdot H_2O$ (10 mg/mL) (1.25 mg Pd element, equivalent to 7.0 wt% Pd loading) and 10 mL

of CTAB solution (12.5 mM) were added and followed by irradiation from a mercury lamp at (130 V*5 A) W (210 mW/cm^2) for 30 min, supported Pd nanocube seeds were formed on rGO (GO-supported Pd). Turned off the lamp and washed by DI water after the irradiation, 112.6 μL $HAuCl_4{\cdot}4H_2O$ (100 mg/mL) (0.54 mg Au element, equivalent to 3.0 wt% Au loading) was added, and then the solution was further illuminated by a mercury lamp at (130 V*5 A) W (210 mW/cm^2) for 30 min, and followed by a mercury lamp at (130 V*8 A) W (360 mW/cm^2) for 90 min. The black solid of Pd cube-Au HNCs was collected through a filtration and drying process.

Characterization

The transmission electron microscopy (TEM), High-resolution TEM (HRTEM) and scan TEM characterizations were performed on JEOL JEM-2010F, 2100F and spherical aberration corrected Transmission Electron Microscope (ACTEM) at 200 kV. Energy dispersive spectroscopy (EDS) mappings were conducted in STEM mode equipped with a single drift detector (X-Max N, OXFFORD Energy Dispersive Spectrum, Oxford 80 SDD). The X-ray diffraction (XRD) patterns were recorded using X-ray diffractometer with Kα1 radiation (Rigaku smartlab) operated at 9 kW (20-45 kV and 10-200 mA) and a secondary beam graphite monochromator from 10° to 90° (2θ), sweep at 10°/min. X-ray photoelectron spectroscopy (XPS) measurements were conducted using the K-Alpha+ XPS system (Thermo Kalpha) and the X-ray source was Al Kα radiation. The XPS data were internally standardized with respect to the C 1s peak position at 284.8 eV. Inductively coupled plasma optical emission spectrometry (ICP-OES) was performed on a PerkinElmer 8300. Zeta potential was analyzed on Malvern zeta potentiometer (Zetasizer Pro). Zeta potential was tested by Nano Sizer and Zeta-potential Tester) (Zetasizer Nano ZS, Malvern).

Amplitude Modulation-Kelvin Probe Force Microscopy (AM-KPFM) measurement.

During KPFM measurements, the contact potential difference (CPD) signals between the probe and samples were measured by amplitude-modulated mode with a Bruker SCM-PIT-V2 probe under ambient conditions. For denoting the surface potential imaging, the lift height was adjusted to 50 nm to obtain the best signal-to-noise ratio and minimize the possible cross-talk effect during the KPFM imaging measurement. Surface photovoltage microscopy (SPVM) refers to the change of the CPD before and after illumination with the same tip, which can be defined as $SPV = \Delta CPD = CPD_{light} - CPD_{dark}$, where the CPD_{light} and CPD_{dark} were characterized by SPVM through the continuously mapping the surface potential images in the dark and under illumination using KPFM, respectively. The SPV light source consists of a 300W xenon lamp (PLS-SXE300, Beijing Perfectlight Co.Ltd) and an Omni-λ 500 monochromator.

Evaluation of electrocatalytic activity

Preparation of Electrodes.

Gas-diffusion-electrodes (GDEs) were prepared via spraying catalyst slurry onto a gas-diffusion-layer (GDL, H14C9, Freudenberg) by airbrush technique. To ensure a well-dispersed catalyst slurry, 20 mg of catalyst, 3 mL of isopropanol, and 30 μL of Nafion solution (Dupont, D-520 dispersion, 5% w/w in water and 1-propanol, Alfa Aesar) were mixed and ultrasonic dispersed for 1 hour. Afterward, the catalyst slurry was carefully sprayed onto a GDL to achieve a catalyst loading of ~ 0.5 mg·cm^{-2}. The actual catalyst loading was determined by weighing the GDL before and after spraying. For cathode GDE, the catalysts were our prepared materials. For anode GDE, commercial IrO_2 nanoparticles were used as catalysts [S2].

Electrochemical Measurements.

The CO_2RR performance was measured in a flow cell, which was comprised of a catalyst-sprayed GDE as the cathode, an IrO_2-sprayed GDE as the anode, and an anion exchange membrane (FAB-PK-130, Fumatech) to separate the cathode and anode chambers. A peristaltic pump was used to deliver the alkaline electrolyte (1 M KOH) through both cathodic and anodic compartments at 10 $mL \cdot min^{-1}$. A mass flow controller (DSN MFC-700, Designtech) was used to deliver CO_2 into the gas chamber at a constant flow rate of 40 $mL \cdot min^{-1}$. The flow channels in both anode and cathode compartments were 2 cm × 0.5 cm. The cathode GDE and anode GDE were both clipped to 2.5 cm × 1 cm. The anion exchange membrane was 2.5 cm × 1 cm. All the electrochemical experiments were conducted using an electrochemical workstation (Gamry Instruments, Reference 3000) with an Ag/AgCl reference electrode. Electrode potentials were rescaled to the reversible hydrogen electrode (RHE) reference by:

$$E\ (vs. RHE) = E\ (vs. Ag/AgCl) + 0.197\ V + 0.0591 \times pH \tag{S1}$$

All the measure potentials in this work did not have iR correction [S3].

CO_2RR Product Analysis.

The CO_2RR products were quantified over a period of 600 s at each potential. The gas-phase products were quantified by an online gas chromatograph equipped with a thermal conductivity detector and a flame ionization detector (Shanghai Ramiin GC 2060). Liquid products were quantified by ^1H nuclear magnetic resonance (NMR) spectroscopy (Bruker AVANCE III HD 500 MHz). The pre-saturation method was used to suppress the water peak.

Supporting Figure S

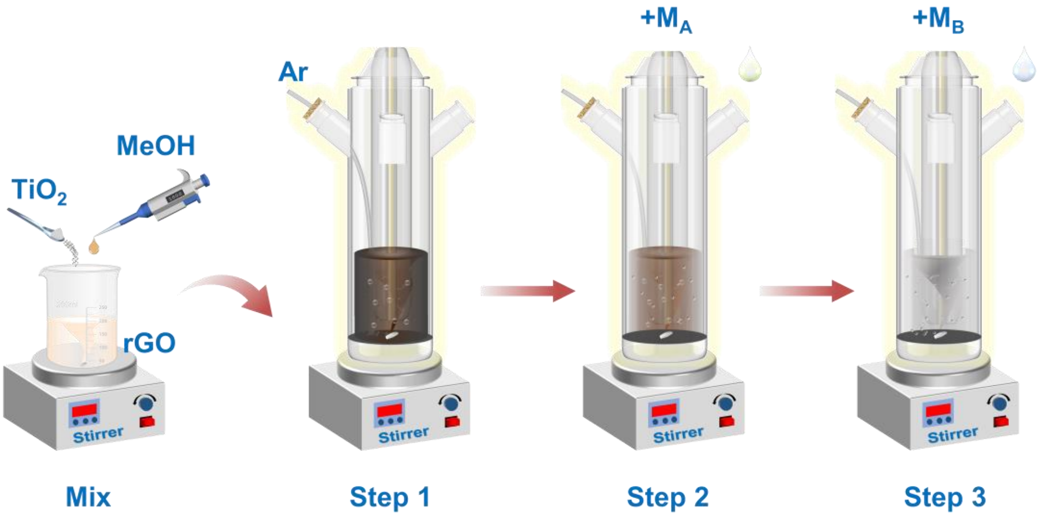

Figure S1. Schematic illustration of the preparation process.

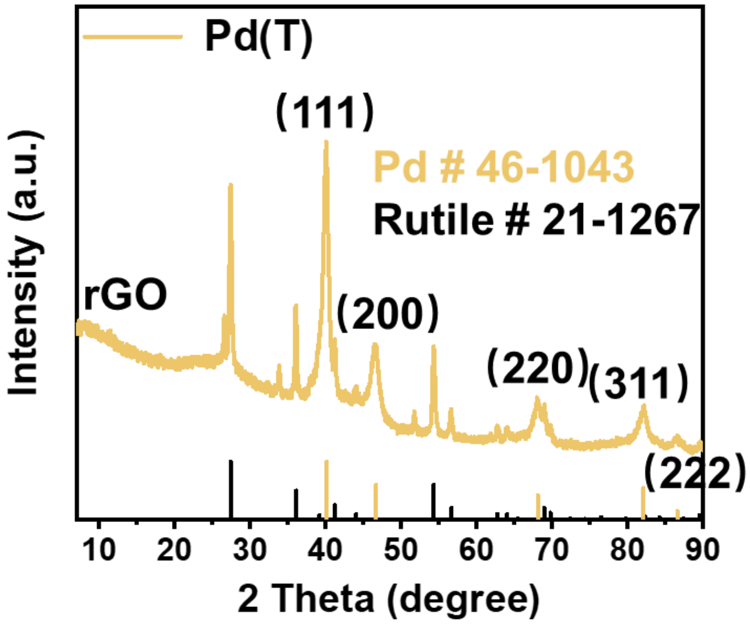

Figure S2. XRD pattern of supported Pd(T) HNCs.

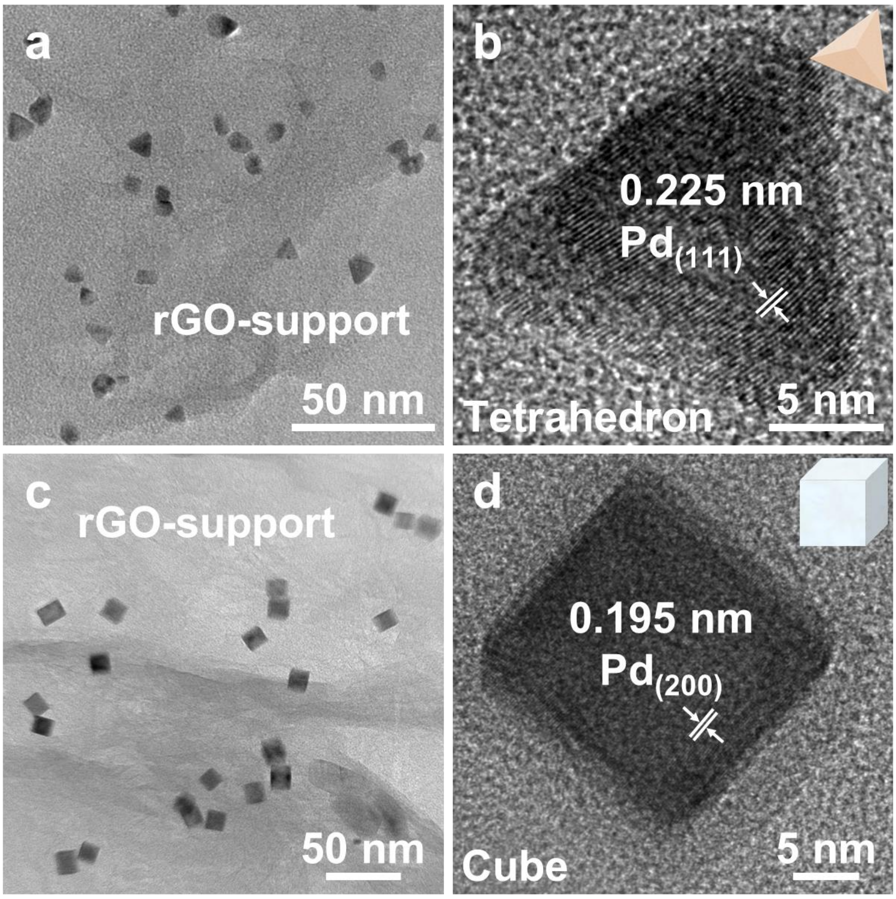

Figure S3. TEM and HRTEM of (**a**,**b**) supported Pd(T) and (**c**,**d**) Pd cube.

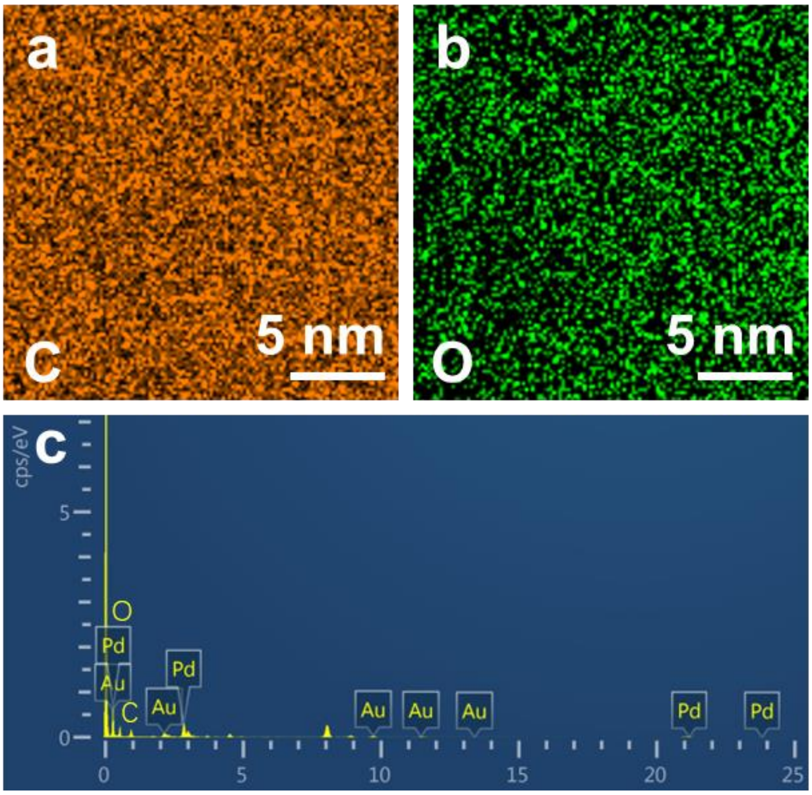

Figure S4. EDS elemental mapping images of (**a**) C, (**b**) O, and (**c**) atomic ratio EDS spectra of Pd(T)@Au.

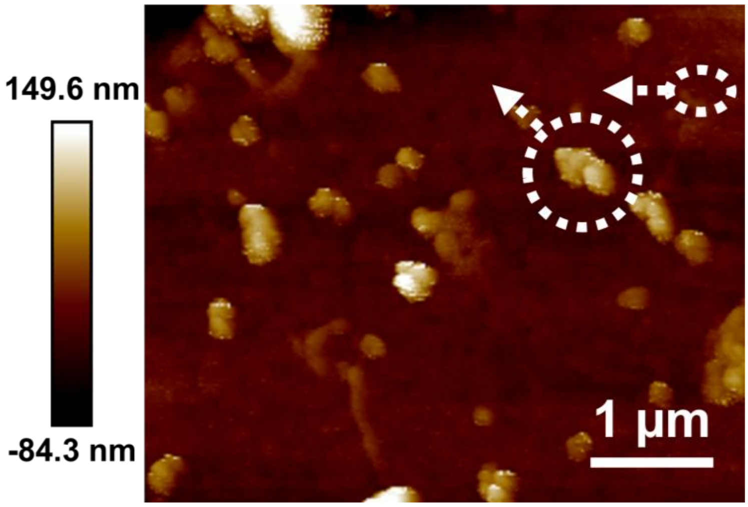

Figure S5. AFM topography of supported Pd seeds.

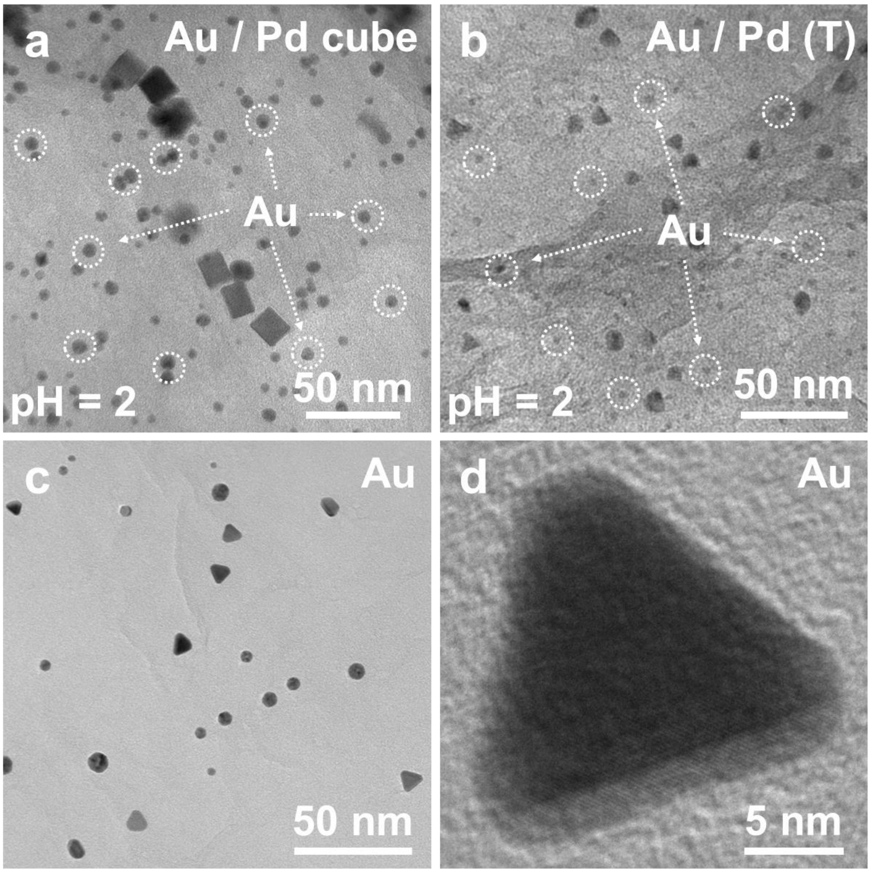

Figure S6. TEM images of supported (**a**) Au / Pd cube and (**b**) Au / Pd(T). (**c**) TEM and (**d**) HRTEM of supported Au.

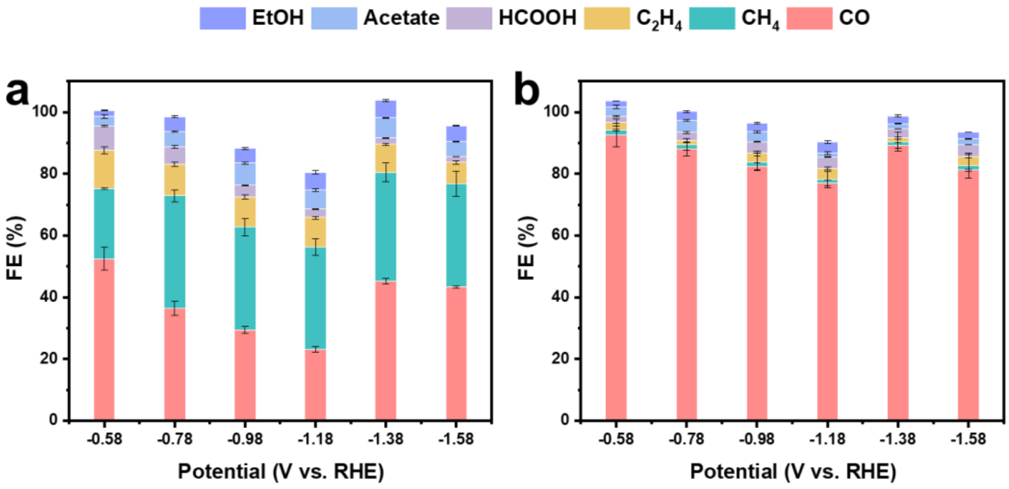

Figure S7. Average FEs of CO_2RR products over (**a**) Pd(T)@Au and (**b**) Pd cube-Au at different potentials.

Supporting Tables

Table S1. Abbreviation names of the samples.

Samples	Abbreviation Name
Pd tetrahedron	Pd(T)
Pd nanocube	Pd cube
Pd tetrahedron @ Au nanocrystals	Pd(T)@Au
Pd nanocube - Au nanocrystals	Pd cube-Au
Supported Au nanocrystals + supported Pd nanocube	Au / Pd cube
Supported Au nanocrystals + supported Pd tetrahedron	Au / Pd(T)

Note: The filtrate obtained after the photodeposition reaction was tested by ICE-OES and the results revealed the absence of Pd and Au, indicating the complete reduction of all precursors. Moreover, the synthesized material was dissolved to determine the content of Pd and Au.

Table S2. Zeta potentials of supported Pd seeds at different pH values in solution.

pH value	Zeta potential (mV)
6.0	-25.6
5.5	-18.7
5.0	-16.0
4.5	-12.6
4.0	-8.1
3.5	-3.9
3.0	-0.1
2.5	1.5
2.0	3.7
1.5	5.2
1.0	7.0
0.5	9.9

Note: The initial solution, containing TiO_2-rGO-supported Pd, had a pH value of approximately 5.0. Furthermore, it was observed that the zeta potential of the solution became positive when the pH value dropped below 3.0.

Table S3. Total current densities on supported HNCs at different fixed potentials.

Potential (V vs. RHE)	Total current density of supported Pd(T)@Au HNCs ($mA \cdot cm^{-2}$)	Total current density of supported Pd cube-Au HNCs ($mA \cdot cm^{-2}$)	Total current density of supported Au/Pd cube HNCs ($mA \cdot cm^{-2}$)
-0.58	-62	-71	-55
-0.78	-149	-200	-110
-0.98	-261	-350	-199
-1.18	-452	-570	-375
-1.38	-698	-903	-526
-1.58	-1010	-1298	-768
-1.78	-1399	-1805	-921

Table S4. CO FEs on supported HNCs at different fixed potentials.

Potential (V vs. RHE)	CO FEs of supported Pd(T)@Au HNCs (%)	CO FEs of supported Pd cube-Au HNCs (%)	CO FEs of supported Au/Pd cube HNCs (%)
-0.58	52.6 ± 3.2	92.6 ± 3.5	77.1 ± 7.0
-0.78	36.4 ± 1.9	88.1 ± 3.1	69.0 ± 6.6
-0.98	29.5 ± 2.9	82.5 ± 2.9	57.4 ± 7.4
-1.18	23.1 ± 2.7	77.0 ± 5.5	45.7 ± 8.0
-1.38	45.2 ± 3.0	89.2 ± 1.2	55.6 ± 5.7
-1.58	43.3 ± 4.1	81.3 ± 2.2	52.3 ± 6.8

Table S5. CO current densities (Partial current density) on supported HNCs at different fixed potentials.

Potential (V vs. RHE)	Partial current density of supported Pd(T)@Au HNCs (mA·cm^{-2})	Partial current density of supported Pd cube-Au HNCs (mA·cm^{-2})	Partial current density of supported Au/Pd cube HNCs (mA·cm^{-2})
-0.58	-31.5	-66.1	-42.4
-0.78	-54.7	-176.2	-75.9
-0.98	-76.3	-288.8	-114.2
-1.18	-104.1	-438.9	-171.8
-1.38	-316.5	-802.8	-292.2
-1.58	-438.2	-1056.9	-400.5

References

S1. Marcano, D. C.; Kosynkin, D. V.; Berlin, J. M.; Sinitskii, A.; Sun, Z.; Slesarev, A.; Alemany, L. B.; Lu, W.; Tour, J. M. Improved synthesis of graphene oxide. *ACS Nano* **2010,** *4*, 4806-4814.

S2. Liu, Y.; Ji, Y.; Li, Q.; Zhu, Y.; Peng, J.; Jia, R.; Lai, Z.; Shi, L.; Fan, F.; Zheng, G.; Huang, L.; Li, C. A surfactant-free and general strategy for the synthesis of bimetallic core–shell nanocrystals on reduced graphene oxide through targeted photodeposition. *ACS Nano* **2023,** *17*, 15085−15096.

S3. Lv, X.; Liu, Z.; Yang, C.; Ji, Y.; Zheng, G. Tuning structures and microenvironments of Cu-based catalysts for sustainable CO_2 and CO electroreduction. *Accounts of Materials Research* **2023,** *4*, 264−274.
